# Impacts of regular and random noise on the behaviour, growth and development of larval Atlantic cod (*Gadus morhua*)

**DOI:** 10.1098/rspb.2015.1943

**Published:** 2015-10-22

**Authors:** Sophie L. Nedelec, Stephen D. Simpson, Erica L. Morley, Brendan Nedelec, Andrew N. Radford

**Affiliations:** 1 School of Biological Sciences, University of Bristol, Life Sciences Building, 24 Tyndall Avenue, Bristol BS8 1TQ, UK; 2 USR 3278 CRIOBE CNRS-EPHE-UPVD, CRIOBE BP 1013 Moorea, 98729 Polynesie Francaise, UK; 3 Biosciences, College of Life and Environmental Sciences, University of Exeter, Stocker Road, Exeter EX4 4QD, UK

**Keywords:** anthropogenic noise, regularity, developmental stages, tank experiments, fish

## Abstract

Anthropogenic noise impacts behaviour and physiology in many species, but responses could change with repeat exposures. As repeat exposures can vary in regularity, identifying regimes with less impact is important for regulation. We use a 16-day split-brood experiment to compare effects of regular and random acoustic noise (playbacks of recordings of ships), relative to ambient-noise controls, on behaviour, growth and development of larval Atlantic cod (*Gadus morhua*). Short-term noise caused startle responses in newly hatched fish, irrespective of rearing noise. Two days of both regular and random noise regimes reduced growth, while regular noise led to faster yolk sac use. After 16 days, growth in all three sound treatments converged, although fish exposed to regular noise had lower body width–length ratios. Larvae with lower body width–length ratios were easier to catch in a predator-avoidance experiment. Our results demonstrate that the timing of acoustic disturbances can impact survival-related measures during development. Much current work focuses on sound levels, but future studies should consider the role of noise regularity and its importance for noise management and mitigation measures.

## 1. Introduction

Some anthropogenic (man-made) noise, such as that arising from traffic, resource extraction and construction, is now recognized as pollution both in air and underwater [1,2]. From individual behaviour and physiology up to community structure, a wide variety of species are affected by noise [3,4]. However, the majority of experiments have examined the impact of short-term exposure [5,6]. Repeated and/or chronic exposure could alter how terrestrial and aquatic animals respond to noise as a consequence of changes across time and cumulative effects [7–9]. Recent evidence using brief (30 min) exposures also indicates that different temporal patterns of noise may impact animals in different ways [10], but long-term studies of how different noise patterns or ‘regimes’ may affect animals differently are needed for more effective regulation of this global pollutant.

When exposure to any stressor (physical, chemical or perceived) is repeated, animals could either habituate (where responses diminish with repeat exposures due to increased tolerance) or sensitize (where responses augment due to reduced tolerance) [7]. Shifts in tolerance may be dependent on the intensity, duration and interval time of stressors (reviewed in [11]). In humans, unwanted repetitive sound can become annoying and disrupt task performance, especially if noise is irregular (reviewed in [12]). Regularity of noise does not affect cognitive impairment in rats [13], but stress responses in fish can be influenced by regularity in other contexts; for example, regular confinement leads to a reduced cortisol (stress) response compared with irregular confinement in the cichlid *Oreochromis mossambicus* [14]. Knowledge about the impacts of regular compared with random noise is important in the context of regulation, because patterns of activity could be altered to minimize effects of anthropogenic noise.

We examine how repeated exposure to regular and random acoustic disturbance (playback of recordings of ship noise) during rearing affects behaviour, growth and body-shape development in larval Atlantic cod (*Gadus morhua*). Previous studies on impacts of anthropogenic noise on aquatic organisms have focused on behaviour and physiology (e.g. [9,15–17]), with changes during development understudied. Young animals may be most vulnerable due to reduced ability to move away from sources of noise. Noise has been shown to cause body malformations and delay development in scallop embryos [18], impair survival of embryos and the growth of larvae in fish [19], and compromise embryonic development and larval survival in sea hares [20]. Effects on survival during early life stages when natural mortality is high can result in greater population fluctuations than impacts at the adult stage [21], and survival through developmental stages is a key driver of population dynamics.

Due to their socio-economic importance and the vulnerability of many species to anthropogenic pressures such as overfishing and climate change [22,23], fish are an important taxon to consider with respect to acoustic noise. All fish detect sound, often possessing specialized auditory apparatus, and thus are exposed to underwater anthropogenic noise, including ships across the globe [24,25]. Mounting evidence shows that at least some fish species can be negatively impacted by noise (e.g. [15–17,26]), but whether these effects persist with repeated exposure is unknown. We studied Atlantic cod because of their auditory ability [27], high socioeconomic value, vulnerability to overfishing and north Atlantic distribution, which overlaps with one of the busiest shipping areas in the world [28,29].

We reared cod from hatching in three different noise regimes: continuous playback of ambient harbour noise; regular additional noise (continuous playback of ambient harbour noise plus recordings of ships passing through the harbour played back in a regular pattern); and random additional noise (continuous playback of ambient harbour noise plus the same recordings of ships played back in a random pattern). We predicted that exposure to additional noise during rearing would reduce growth, increase yolk sac use and reduce body width–length ratio (condition indicator), and that these responses would be lessened by habituation when noise exposure during rearing was regular but not when random. We also predicted that short-term exposure to additional noise would lead to increased startles and reduced predator-avoidance behaviour, with these behavioural responses lessened by habituation in fish that had been reared while exposed to regular additional noise compared with fish reared in control conditions.

## 2. Material and methods

Work was carried at Ardtoe Marine Laboratories, Acharacle, West Highlands, Scotland. Twelve tanks were allocated randomly across the three treatments: control ambient noise (‘A’), regular additional noise (‘R’), random additional noise (‘Rand’). Hatching-stage cod eggs from four separate batches obtained from broodstock (see the electronic supplementary material for rearing protocol and tank details) were allocated to treatments in the most balanced way possible (given a stocking density of 7000 eggs per tank): one batch was split between two treatments (A, R); two batches were split between all three treatments (A, R, Rand); and the final batch was split between the remaining four tanks (A, R, Rand, Rand).

Sound exposure began 6 h after eggs hatched and continued 24 h per day until the end of the experiment, after sampling at 16 days post-hatching (dph). We refer here to ‘playback of ambient noise’ and ‘playback of ship noise’ to mean introduction of sound using acoustic recordings of ambient noise and ship noise via loudspeakers. The sound exposures we used were: ambient control (playback of ambient noise 24 h per day); regular additional noise (playback of ambient noise with one 15-min ship pass per hour); and random additional noise (playback of ambient noise with six 15 min ship passes every 6 h at random times, allowing for overlapping). The ‘traffic exposure’ for regular and random treatments was thus the same over any 6 h period. Electronic supplementary material, figure S1, shows example sound-pressure and particle-acceleration levels in rearing tanks. Four different replicates of each sound treatment were used (one per tank). Details on playback construction are in the electronic supplementary material.

### (a) Startle response at 12 h post-hatching

Preliminary observations revealed that newly hatched fish were either still or startling (rapid contractions of muscles causing body curvature) and that they ‘settled’ (when the startle responses reached a stable baseline rate of 1–2 per min) within 2 min of disturbance (after introduction to the arena and after acoustic disturbance). A repeated-measures experiment was conducted to test how individual fish (six from each rearing tank) responded to short-term exposure to an additional-noise (ship recording) track or a matching control (ambient noise) track originating from the same harbour. Each fish (measuring approx. 5 mm) was introduced to the experimental arena (a Petri dish containing new water for each trial, with opaque bottom and sides suspended 10 cm above a loudspeaker in a bucket of water 25 cm deep), allowed to settle for 2 min, and then exposed to one of the playback tracks. After 2 min re-settling time, the fish received the second playback track. During treatments, the number of startles was counted. All observations were made by S.L.N., who was blind to the rearing condition of fish. Five different additional-noise and control tracks were used and the order of treatments was balanced. Sound-pressure levels of additional-noise and control playbacks were measured (electronic supplementary material, figure S1c); due to the size of the experimental arena, it was not possible to measure particle acceleration.

### (b) Growth: use of yolk sac, size-at-age and body width–length ratio

Photographs were taken of five to 10 fish from each rearing bin at 1 dph (before first feed), 2 dph (after first feed) and 16 dph, under a microscope with 10 mm graticule connected to a digital camera. One bin from each treatment could not be sampled at day 16 due to low survival. The maximum length and width measures of the yolk sac were digitized using four landmarks via TpsDig software [30]. Yolk sac centroid size (a metric of size calculated as the square-root of the sum of squared distances of individual landmarks from the centroid of the landmark configuration [31]) was determined using TpsRelw [32]. Body length was digitized using six landmarks from the tip of the top lip to the base of the tail, and myotome length was digitized in TpsDig and PAST [32] by two landmarks either side of the myotome at the position of the anus (electronic supplementary material, figure S2). Myotome length is a measure of the amount of muscle on the fish. Body width–length ratio was calculated as myotome length divided by body length.

### (c) Anti-predator response at 16 days post-hatching

We developed an independent-measures anti-predator response experiment, whereby flight behaviour was assessed in response to attempts to catch the fish using a pipette (the same method used for transferring fish). We used the same arena as for the startle-response experiment. Ten individuals from each rearing tank were tested. Larvae were allowed 4 min settling time during which time ‘flight responses’ (swimming rapidly in any direction) ceased in all cases within the first 2 min. Fish were then exposed to 3 min playback of either a control (ambient harbour) track or an additional-noise (ship recording) track, the order of which (between fish) was randomized and controlled by an assistant. After 3 min of playback, the fish was approached with a 1 ml pipette from behind and chased until it was caught in the pipette. The response measure was thus ‘time-to-catch’. All pipette manipulations were made by S.L.N., who was blind to the rearing condition of the fish and to the test sound treatment due to masking by music through earphones (see also [17]). Sound-pressure levels of recordings of control and additional-noise conditions in the experimental arena were measured (electronic supplementary material, figure S1d).

### (d) Statistical methods

General linear mixed-effects models (LMMs) fitted by maximum likelihood (Laplace approximation) were used, where distributions of data allowed sufficiently good model fit (after log transformation to meet the assumption of normality where necessary), to test for the effects of noise treatment while controlling for the random effects of rearing bin and batch. See the electronic supplementary material for description of how these tests are used. Rearing noise treatment (ambient, regular, random), short-term playback (control, additional noise) and dph were included as fixed effects.

Startle response data were distributed in a way that precluded general or generalized LMMs fitting the data well. In this case, a Wilcoxon signed-ranks test was used to test the effect of short-term playback on the number of startles made by an individual. An ANOVA was used to test the effect of rearing noise treatment on the log-transformed difference in the number of startles in ambient versus ship-noise playback within individual fish. All statistics were performed in R v. 3.0.1.

## 3. Results

### (a) Startle response at 12 h post-hatching

**Figure 1.**
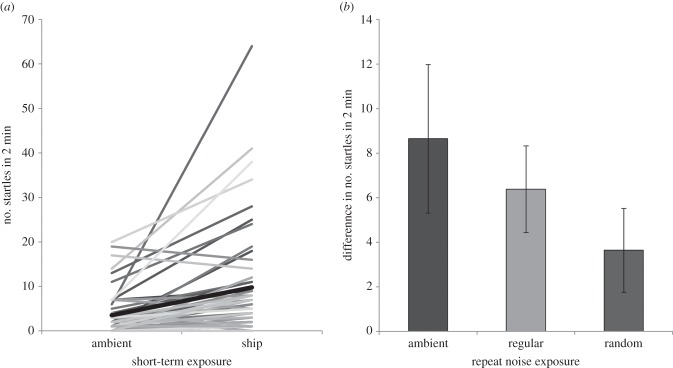
Startle responses of larval cod. (*a*) Median number of startles during 2 min exposure to ambient and additional-noise playbacks represented by black line. Other grey lines join results for individual fish in each treatment. *n* = 52. (*b*) Mean ± 1 s.e. difference in number of startles in additional-noise playback compared with ambient-noise playback for fish from the three different rearing noise treatments. *n* = 17–18 per rearing treatment.

Cod larvae startled significantly more often (a median of 4.5 more startles in a 2 min period) when exposed to short-term additional noise compared with a control playback (Wilcoxon test: *W* = 758.5, *n* = 52, *p* < 0.001; figure 1*a*). The startle responses began at the onset of experimental additional noise and continued intermittently throughout the 2 min of playback. There was no significant effect of rearing noise treatment on the difference between the number of startles in the two short-term playback trials (ANOVA: *F*_2,49_ = 1.49, *p* = 0.235; figure 1*b*).

### (b) Use of yolk sac

**Figure 2.**
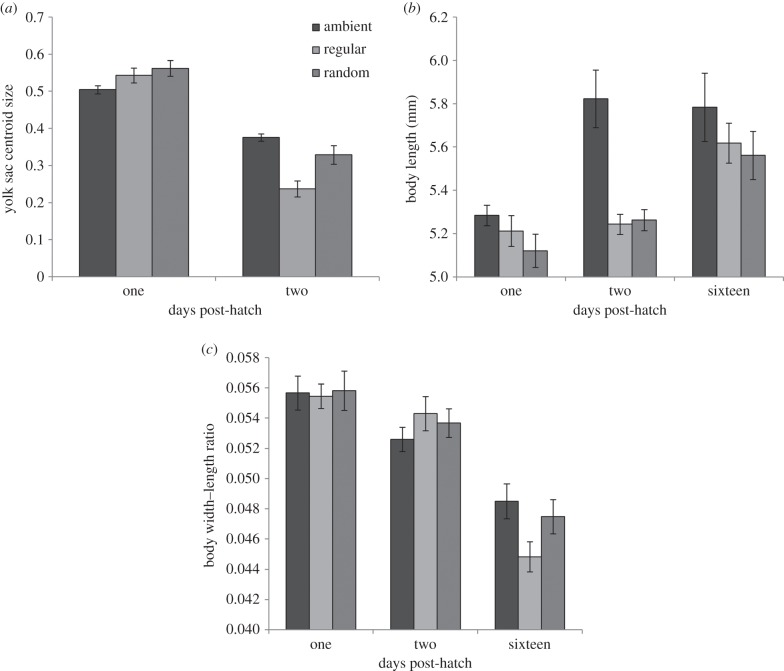
(*a*) Mean ± 1 s.e. yolk sac centroid size (unitless measure) at 1 and 2 dph. (*b*) Mean ± 1 s.e. body length at 1, 2 and 16 dph. (*c*) Mean ± 1 s.e. body width–length ratio (myotome length/body length) at days 1, 2 and 16 post-hatching. *n* = 19–35 per treatment/day combination.

After controlling for effects of rearing bin (LMM: variance = 0.002, s.d. = 0.048) and batch (variance = 0.004, s.d. = 0.059), yolk sac centroid size was significantly affected by the interaction between rearing noise treatment and dph (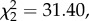 *p* < 0.001; rearing noise treatment: 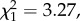 *p* = 0.195; dph: 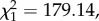 *p* < 0.001; *n* = 25–35 per treatment/day combination; figure 2*a*). Overall, yolk sacs decreased in size between days 1 and 2 by 0.128 ± 0.022, but fish reared with regular additional noise had yolk sacs at day 2 that were smaller than those in the control (*t*-test: *t*_232_ = 3.53, *p* = 0.001; effect size = 0.148, s.e. = 0.042) and random (*t*_232_ = 2.31, *p* = 0.021; effect size = 0.094, s.e. = 0.041) treatments; yolk sacs in random and control treatments were not significantly different in size at day 2 (*t*_232_ = 1.30, *p* = 0.194; effect size = 0.054, s.e. = 0.041).

### (c) Size-at-age

After controlling for bin (LMM: variance < 0.001, s.d. < 0.001) and batch (variance < 0.001, s.d. < 0.001), there was a significant interaction between rearing noise treatment and dph on size-at-age (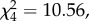 *p* = 0.032; rearing noise treatment: 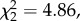 *p* = 0.089; dph: 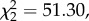 *p* < 0.01; *n* = 19–35 per treatment/day combination). Fish from all three rearing conditions grew during the 16-day experiment (figure 2*b*), but at 2 dph, fish from the control treatment were longer than those from both regular and random noise treatments (control cf. regular: *t*_250_ = 2.68, *p* = 0.008; control cf. random: *t*_250_ = 2.68, *p* = 0.008), which did not differ significantly from one another (regular cf. random: *t*_250_ = 0.01, *p* = 0.990). There was no significant difference between lengths of fish from different rearing noise treatments at day 16 (see electronic supplementary material, table S2, for results of all planned contrasts).

### (d) Body width–length ratio

After controlling for bin (LMM: variance < 0.001, s.d. = 0.002) and batch (variance < 0.001, s.d. = 0.001), there was a non-significant trend for an effect of the interaction between rearing noise treatment and dph on body width–length ratio (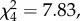 *p* = 0.098; rearing noise treatment: 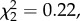 *p* = 0.898; dph: 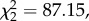 *p* < 0.001; *n* = 21–35 per treatment/day combination; figure 2*c*). Overall, width–length ratio declined during the course of the experiment, but the greatest decline was in fish from the regular noise treatment, leading them to be significantly different from controls at 16 dph (*t*_265_ = −1.98, *p* = 0.049). There was no significant difference in width–length ratio between fish from different rearing noise treatments at day 2 (see electronic supplementary material, table S3, for results of all planned contrasts).

### (e) Anti-predator response at 16 days post-hatching

**Figure 3.**
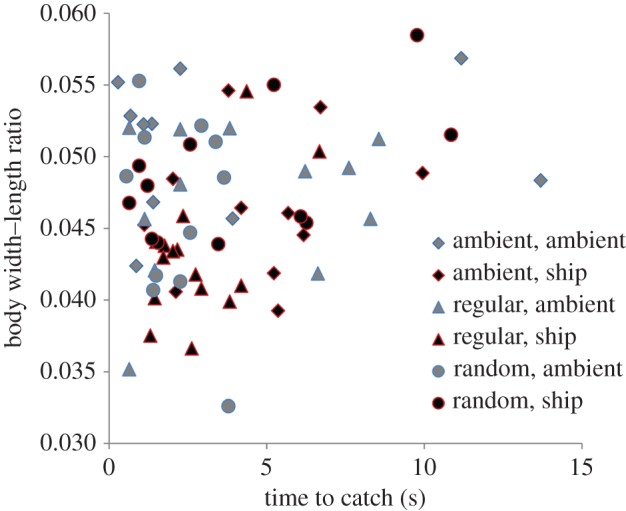
Time taken to catch fish with a pipette depending on body width–length ratio. Data points are coded according to rearing noise treatment (shape) and short-term noise exposure (grey/black). *n* = 13–17 per rearing treatment/short-term noise treatment combination. (Online version in colour.)

After controlling for bin (LMM: variance = 0, s.d. = 0) and batch (variance = 0.005, s.d. = 0.070), there was a non-significant trend towards an effect of short-term noise exposure on time to catch (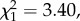 *p* = 0.065; figure 3). Fish took 0.17 ± 0.09 s longer to be caught during additional-noise playback compared with ambient-noise playback. Rearing noise treatment did not significantly affect time to capture (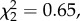 *p* = 0.724). We investigated the relationship between noise, morphology and behaviour *post hoc* and found that width–length ratio had a significant effect on time to catch (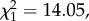 *p* < 0.001, *n* = 13–17 per rearing treatment/short-term noise treatment combination; figure 3). An increase in width–length ratio of 0.1 meant fish took 0.9 ± 0.8 s longer to be caught.

## 4. Discussion

Exposure to additional acoustic noise affected larval cod behaviour, growth and development. Short-term exposure caused startle responses in newly hatched larvae. Two days of additional noise of both regular and random regimes reduced growth, while regular noise led to faster yolk sac use. After 16 days, growth converged, although fish exposed to regular noise had lower body width–length ratios. Larvae that had a lower body width–length ratio were easier to catch in a predator-avoidance experiment. Although noise regime during rearing did not directly affect the behaviours measured, regular noise could impact larval cod survival via an indirect effect on body development. Other studies have found mixed results on effects of noise on growth in fish [19,33–35]. We provide the first evidence of an effect of anthropogenic noise on larval yolk sac use. Moreover, we demonstrate that noise regime can affect impacts (see also [10]). Our results were contrary to our hypothesis that a random regime would be worse than a regular one, as was found in relation to other stressors in fish [14]; rather, regular noise was more disturbing than random noise.

Newly hatched fish startled more often during additional noise than controls in the short term. Noise-induced startle responses have been reported in adult fish by other researchers (e.g. [27,36]). Six hours prior exposure to regular or random noise did not affect the tolerance of larvae to noise in the short-term experiment, suggesting neither habituation nor sensitization. As noise is not a direct threat of predation, startling during noise with failure to habituate may incur energetic costs to larvae without any associated fitness benefits.

Larvae exposed to regular and random noise grew less between days 1 and 2 than ambient controls, but growth caught up by day 16. Banner & Hyatt [19] found that fish larvae exposed to higher noise levels grew less in the first 12 dph, while Bruintjes & Radford [34] found that noise did not impact larval fish length or weight after four weeks post-hatch. Similarly, Davidson *et al*. [33] found that higher noise levels reduced juvenile growth in the first month followed by catch-up growth, resulting in no difference after five months. Stunted initial growth could be an indicator that noise is a stressor [11]. Subsequent catch-up growth could lead to lower lifetime fitness due to oxidative stress, as has been previously shown in fish [37].

Larvae exposed to regular noise used their yolk sacs faster after 2 days of exposure and had a lower body width–length ratio after 16 dph compared with those raised in ambient or random noise. Lower body width–length ratio suggests less muscle per body size. Regular noise may lead to a shift in resource allocation from maintenance of reserves to chronic activation of the adrenal system, incurring an allostatic load [38]. Alternatively, larvae may have perceived additional noise as a source of risk, diverting attention towards risk detection and avoidance, reducing foraging efficiency [36]. After exposure to a source of risk, animals are likely to return gradually, rather than immediately, to a situation where the risk is no longer perceived as relevant [39]. While immediate behavioural responses such as startles may quickly return to baseline levels, foraging behaviour is likely to have a longer latency for recovery. It is therefore possible that the time intervals between regular additional-noise events (45 min) did not allow time for sufficient recovery of foraging behaviour to compensate for the energetic costs when foraging was disrupted. This may have led to a cumulative stress response [40].

There was a trend towards short-term playback of additional noise leading to fish taking longer to catch, which contrasts with previous results showing the impacts of noise on predator-avoidance behaviour [17]. However, this effect was less strong than the effect of body width–length ratio. Larvae with lower body width–length ratios were caught faster in the predator-avoidance experiment. We did not find a direct effect of rearing noise treatment on time-to-catch, but our results suggest that regular noise exposure could indirectly affect survival via an effect on body width–length ratio. An effect on survival at this early life-history stage, even if subtle, may have consequences for population dynamics because high mortality of the early stages means that small changes in selective mortality have a substantial influence on population fluctuations [21].

Fish larvae in regular and random regimes were exposed to the same number of playbacks of ship recordings on average (six every 6 h), but the regular regime had a stronger effect than the random regime. The random treatment included both shorter and longer time intervals than the regular disturbance. We hypothesize that shorter time intervals during the random disturbance had no further impact, while longer time intervals during random disturbance allowed compensation and/or habituation (many species of fish show their highest plasma cortisol levels within 0.5–1 h after a stressful disturbance [11]). It is also possible that the greater intensity of sound occurring when two additional-noise incidences overlapped in time had no further impact, while the reduction in total time of additional-noise exposure brought about by such overlaps contributed to the longer time intervals allowing compensation and/or habituation. Therefore, further work could potentially reveal that regular disturbance with longer time intervals between exposures than in this experiment may result in reduced effects on yolk sac use, growth and development.

We used underwater loudspeakers to expose the larvae to noise in tanks, and this is not fully representative of anthropogenic noise in natural settings; due to proximity to the sound source, the particle motion component of the sound was higher than would be expected for comparable pressures in natural conditions where ships were passing. Interference of sound waves due to reflections from tank boundaries and the frequency response of speakers also meant that some frequencies were comparatively louder or quieter than would be expected of real ship or ambient harbour noise. It should also be noted that the acoustic conditions in the Petri dish experiments would be different from those in rearing tanks (for instance, particle motion would be higher). The importance of our experiments is that they demonstrate the potential for regular and random acoustic disturbances to have different effects, even when the number of additional-noise exposures was carefully controlled. Thus, the use of laboratory conditions allowed us to test for specific effects of disturbance regularity by controlling for potential confounding factors [17]; future work will need to examine how wild fish respond to real-world noise sources in natural conditions. Taken together, our findings reveal that noise can have effects on fish that extend beyond immediate impacts and are dependent on exposure regime. These results therefore have important wider implications for research on the impacts of anthropogenic disturbances on animals.

## Supplementary Material

Impacts of regular and random noise on the behaviour, growth and development of larval Atlantic cod (Gadus morhua) supplementary material

## Data accessibility

Data available from the Dryad Digital Repository: http://doi.org/10.5061/dryad.v30kv.
