# Supplementary material for: Impacts of regular and random noise on the behaviour, growth and development of larval Atlantic cod (Gadus morhua)
Source: Proc Biol Sci. 2015 Oct 22;282(1817):20151943. doi: 10.1098/rspb.2015.1943 (PMC4633878; doi:10.1098/rspb.2015.1943)

Impacts of regular and random noise on the behaviour, growth and development of larval Atlantic cod (*Gadus morhua*)

**Sophie L. Nedelec, Stephen D. Simpson, Erica L. Morley, Brendan Nedelec, Andrew N. Radford**

# Supplementary Materials

## Cod rearing protocol

Egg collection and egg and larval rearing followed standard procedures adopted at the Viking Fish Farms for cod. Fertilised eggs were collected in April 2012 from 10 female and six male adult Atlantic cod (F2 hatchery-reared brood stock) that were housed at ambient temperature and photoperiod in a cylindrical tank of diameter 5.3 m and depth 3 m, supplied with filtered sea water pumped from the ocean. Eggs were collected at 15:00 each day and checked for developmental stage under the microscope to ensure eggs were developing synchronously; cod generally spawn in the middle of the night (Engen & Folstad, 1999), thus more than 98% of eggs collected were stage 2 (12–24 h post fertilisation, with ‘many cells’). Eggs were disinfected with Sorgene-5 (2 ml per litre) for 45 s to remove any pathogens accumulated in egg collectors before incubation. All eggs collected on a given day were defined as one ‘batch’. Each batch was incubated in a separate 80 L conical tank with gentle aeration and continuous water flow to maintain an even distribution of eggs in the water column, as is common with pelagic marine fish egg aquaculture (Brown *et al.*, 2003). Incubation temperatures were 9.0–10.3°C (within the optimal range for survival; Brown *et al.*, 2003); salinity was 34–35 ppt (shown to give the highest hatching success; Brown *et al.*, 2003); and eggs were incubated in darkness.

Cod were not exposed to noise as part of the experiment until they hatched as larvae, but each batch of eggs was measured throughout the egg phase to avoid potential differences due to egg quality. The quality was measured immediately after collection as the total weight of all live (floating) eggs, the ratio of live to dead (sinking) eggs, and egg size and fertilisation rate. Egg quality was also monitored during incubation by the estimated weight of live eggs remaining, based on the weight of dead eggs cleaned from the bottom of the tank each day. The highest quality batches of eggs available at the time of stocking (as defined by large batches with the best survival, with fertilized eggs of consistent large size) were selected for the rearing experiments. The developmental stages of eggs were monitored by daily examination of a sub-sample under the microscope. Eggs reached the final stage of embryonic development prior to hatching after 75 degree days (number of days post fertilisation x temperature in °C), at which point the number of eggs was estimated from counting the number of eggs in five 60-ml sub-samples. Eggs were disinfected for a second time with Sorgene-5 (2 ml per litre) for 45 s before dividing eggs into one of 12 100-L experimental tanks at 70 eggs per litre.

Once eggs were in the rearing tanks, water flow to bins was set at 0.1 litres per min, air flow was stopped until all eggs had hatched (day 0 post-hatching) to allow eggs to float at the surface. Eggs that did not hatch and died sank to the bottom of the tank and were siphoned out and weighed. Approximately 50% of eggs in the same batch in all cases hatched overnight on the first night in the bin, playback started the following morning; in all cases, the remaining 50% of eggs hatched or died over the following 48 h. Once all eggs had hatched, water flow was reduced to 0.05 litres per min and 500 ml of algae (Dunalliella sp.) was added to increase the opacity of the water and provide a food source for the cod larvae and their rotifer prey (Sherwood, pers. comm.). This did not differ between treatments. A 12:12 photoperiod was provided with an identical light source above each bin. At day 1 post-hatching, ca. 1 million rotifers fed with commercially available enrichment (Multigain or Ori green) were added to each bin. Following this, ca. 1 million rotifers and 500 ml of algae were added to each bin daily. Aeration ensured a uniform distribution of prey, and skimmers removed oil and debris from the surface of the water once the larvae were no longer floating at the surface (between 2 and 5 days post-hatching). At day 10 post-hatching, water flow was increased to 0.1 litres per min. The temperature was 9.7­–11.6°C and salinity was 34–35 ppt.

## Ship and ambient noise recordings

Recordings were made of 12 different ships, fishing vessels and a tug (hereafter referred to as ‘ships’) in Peterhead and Southampton, UK (see Supplementary Table S1 for details). Recordings in Peterhead were made using an accelerometer (M30, sensitivity frequency dependent between 0.1 and 3 kHz (calibrated by manufacturers), Geospectrum Technologies, Dartmouth, Canada) and an omnidirectional hydrophone (HTI-96-MIN, sensitivity -164.3 dB re 1 V/μPa; frequency range 0.002–30 kHz, High Tech Inc., Gulfport MS) connected to a fully calibrated solid-state recorder (PCM-M10, 44.1 kHz sampling rate, Sony Corporation, Tokyo, Japan; recording level calibrated using pure sine wave signals from a function generator with a measured voltage recorded in line with an oscilloscope). The accelerometer and hydrophone were positioned at a depth of 5 m in water 10–12 m deep, close to the entrance to the harbour, where ships passed at 150–250 m at a speed of 9 knots. Recordings in Southampton were made using an omnidirectional hydrophone (Bruel & Kjaer 8103, sensitivity ‑174.0 dB re 1 V/μPa; frequency range: 0.01–180 kHz). The hydrophone was positioned at a depth of 4–8 m in water 8–16 m deep, 20–40 m from the shore. Ships took 15 min to pass, thus each ship recording contains an approach, pass and departure of a vessel where maximum amplitude occurred around 7.5 min. Ambient noise was also recorded at each location under the same recording conditions when no boats or ships were passing.

## Playbacks

An underwater loudspeaker (UW-30, frequency response 0.1–10 kHz, University Sound, Whitehall, Ohio, USA) was mounted, facing upwards, in the bottom of each bin. Playbacks were from mp3 players (Mini Clip, TopTechDirect, UK) via 40 W amplifiers (Kemo Electronic, Germany). Four unique replicates for each of the three sound treatments (ambient (A), regular additional noise (R), random additional noise (Rand)) were made. We used 12 recordings of separate ship passes that were cropped into 15-min samples containing the approach, pass and departure of the vessel. Each R and Rand replicate included an individual combination of nine ships. In regular treatments R1, R2, R3 and R4, one ship was played every hour along with 45 min of ambient noise from the same day and location. Control treatments A1, A2, A3 and A4 mirrored the regular treatments except 15 min ambient noise from the same day and location was played in place of each ship pass to control for an effect due simply to track changes. Random treatments Rand1, Rand2, Rand3 and Rand4 used the same playback files as the equivalent regular treatments, but the timing and order of ship playbacks was randomised within 6 h blocks via an online random number generator, including the possibility of overlapping. Overlapping resulted in higher sound levels (e.g. the peak level increased by 1.6 dB in a case of full overlap). Twenty-four different 6-hour blocks were made for each replicate and these were played in a different randomised sequence each 6 days.

## Acoustic analysis

Acoustic analyses were performed in MATLAB v2010a. Recordings were split into 1 s windows that were Hamming filtered before performing a Fast-Fourier Transform (FFT) of length 44100 to transform the data into the frequency domain. Data were calibrated according to instrument sensitivity and recorder attenuation, then squared before the mean of all the 1 s values were taken at each frequency within each recording. Data were log-transformed to dB re 1 µPa^2^/Hz for pressure or dB re 1 (µm/s^2^)^2^/Hz for particle acceleration. Sound-pressure levels and particle acceleration of ships at sea and their playback in experimental rearing bins, measured in the middle of the water column, were compared (Fig. S1). Since sound-pressure and particle-acceleration levels vary widely over short distances in small bodies of water, these comparisons are for illustrative purposes only and do not necessarily represent the levels fish were exposed to at different places in the rearing bins.

## Statistical testing

General linear mixed effects models

Any effects such as slight temperature variations between bins were controlled for by the statistical models. The variance caused by and standard deviation of the variance for each random effect are presented alongside the results of models. To establish the best-fitting model, terms were eliminated one by one from a maximal model. Simplified models were compared with more complex ones using maximum likelihood ratio tests that employ chi-square statistics to establish whether a simpler model is significantly worse at explaining the data than a more complex one. If a simpler model is not significantly worse when a term is removed, the simpler model is deemed better and thus the term is dropped. If a simpler model is significantly worse, the term is maintained in the model (Meyer, 1991). The degrees of freedom from maximum likelihood tests presented in the Results of the main paper are the difference between the degrees of freedom of the simpler and the more complex models. All potential interactions of fixed effects were examined and are only presented where their exclusion from the model made the model significantly worse at explaining the data at the level p < 0.10. In the case where interaction terms were included in the best model, planned contrasts were conducted using Markov Chain Monte Carlo methods. Z tests were used for post-hoc tests where the sample size was large (> 20); t tests were used where the sample size was small (< 20). Effect sizes are given with standard errors.

# References

Brown JA, Minkoff G, Puvanendran V 2003 Larviculture of Atlantic cod (*Gadus morhua*): progress, protocols and problems. *Aquaculture* **227**, 357–372.

Engen F, Folstad I 1999 Cod courtship song: a song at the expense of dance? *Canadian J Zool* **77**, 542–550.

Meyer K 1991 Estimating variances and covariances for multivariate animal-models by restricted maximum-likelihood. *Genet Sel Evol* **23**, 67–83.

# Tables

Table S1: Details of ships recorded for use in playback experiments

| **Ship** | **Date recorded** | **Port** | **Location** | **Weight (t)** | **Length**  **(m)** | **Breadth**  **(m)** | **Year built** |
| --- | --- | --- | --- | --- | --- | --- | --- |
| Kyoto Express | 04/04/08 | Southampton | 50° 54’ 11” N  1° 26' 23’’ W | 103800 | 334 | 42 | 2005 |
| Vega Stockholm | 04/04/08 |  |  | 8306 | 129 | 21 | 2006 |
| Verlaine | 14/04/08 |  |  | 2893 | 114 | 13 | 1980 |
| Bibby Topaz + Tug Blue Toon | 31/03/12 | Peterhead | 57° 29’ 47” N  1° 46’ 32” W | 5337 | 105 | 20 | 2007 |
| Troms Capella | 31/03/12 |  |  | 4800 | 88 | 19 | 2011 |
| Grampian Talisman | 31/03/12 |  |  | 3890 | 73 | 17 | 2007 |
| Rockwater 1 + 2 fishing boats | 01/04/12 |  |  | 1530 | 98 | 18 | 1983 |
| Skandi Skansen | 01/04/12 |  |  | 4982 | 109 | 24 | 2011 |
| Northern Pioneer | 31/03/12 |  |  |  |  |  |  |
| UP Jasper | 31/03/12 |  |  | 4900 | 87 | 19 | 2011 |
| Aspen | 01/04/12 |  |  | 3159 | 83 | 12 | 2000 |
| Vest Viking (fishing boat) | 01/04/12 |  |  |  | 63 | 13 |  |

Table S2: Planned contrasts for post-hoc testing of the effect of the interaction between rearing noise treatment and days post-hatching on body length. A = Control, R = Regular, Rand = Random. Significant results are shown in bold.

| **Days Post-hatching** | **Rearing Noise Treatments** | **Estimate** | **Standard Error** | **t value** | **Degrees of Freedom** | **p** |
| --- | --- | --- | --- | --- | --- | --- |
| 1 | A x R | 0.15 | 0.20 | 0.76 | 250 | 0.447 |
| 1 | A x Rand | 0.09 | 0.21 | 0.41 | 250 | 0.680 |
| 1 | R x Rand | -0.07 | 0.21 | -0.32 | 250 | 0.746 |
| **2** | **A x R** | **0.54** | **0.20** | **2.68** | **250** | **0.008** |
| **2** | **A x Rand** | **0.54** | **0.20** | **2.68** | **250** | **0.008** |
| 2 | R x Rand | 0.00 | 0.20 | 0.01 | 250 | 0.990 |
| 16 | A x R | 0.34 | 0.22 | 1.55 | 250 | 0.123 |
| 16 | A x Rand | 0.35 | 0.22 | 1.58 | 250 | 0.114 |
| 16 | R x Rand | 0.01 | 0.22 | 0.04 | 250 | 0.972 |

Table S3: Planned contrasts for post-hoc testing of the effect of the interaction between rearing noise treatment and days post-hatching on body width–length ratio. A = Control, R = Regular, Rand = Random. Significant results are shown in bold.

| **Days Post-hatching** | **Rearing Noise Treatments** | **Estimate** | **Standard Error** | **t value** | **Degrees of Freedom** | **p** |
| --- | --- | --- | --- | --- | --- | --- |
| 1 | A x R | -2.59 x 10^-4^ | 1.97 x 10^-3^ | -0.13 | 265 | 0.895 |
| 1 | A x Rand | 5.41 x 10^-4^ | 2.01 x 10^-3^ | 0.27 | 265 | 0.788 |
| 1 | R x Rand | 8.00 x 10^-4^ | 2.01 x 10^-3^ | 0.40 | 265 | 0.691 |
| 2 | A x R | 1.47 x 10^-3^ | 1.99 x 10^-3^ | 0.74 | 265 | 0.459 |
| 2 | A x Rand | 9.89 x 10^-4^ | 2.00 x 10^-3^ | 0.49 | 265 | 0.621 |
| 2 | R x Rand | -4.84 x 10^-4^ | 1.99 x 10^-3^ | -0.24 | 265 | 0.808 |
| **16** | **A x R** | **-4.35 x 10^-3^** | **2.20 x 10^-3^** | **-1.98** | **265** | **0.049** |
| 16 | A x Rand | -3.04 x 10^-3^ | 2.25 x 10^-3^ | -1.35 | 265 | 0.178 |
| 16 | R x Rand | 1.31 x 10^-3^ | 2.18 x 10^-3^ | 0.60 | 265 | 0.547 |

Figure S1 Power spectral density of (a) monoaxial (horizontal axis) particle acceleration and (b) sound-pressure level. Shown are recordings of a ship (10 s at the loudest part of the pass) at sea and its playback in a rearing bin, and of ambient noise (60 s) at sea and its playback in a rearing bin; playback recordings were made 20 cm from the surface in the centre of a rearing bin. Data shown between 0 and 700 Hz as Atlantic cod are thought to be able to hear only up to 400 Hz [42]. (c) Sound-pressure levels of ship, ocean ambient noise and their playback in the startle-response experimental arena. (d) Sound-pressure levels of a ship, ocean ambient noise and their playbacks in the anti-predator experimental arena.


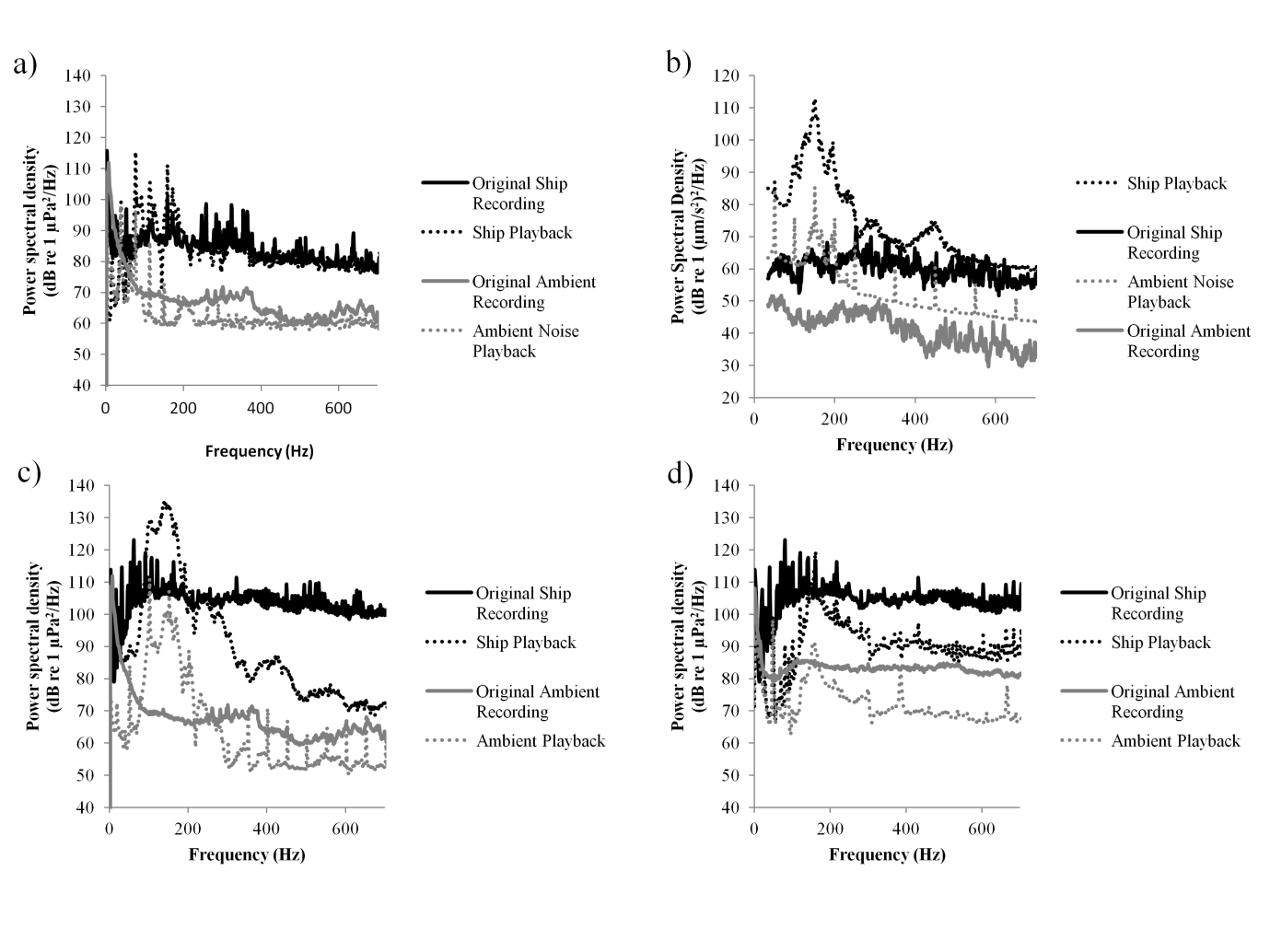


Figure S2 Landmarks digitising yolk sac (1–4), body length (5–10) and myotome length (11–12) in a larval Atlantic cod.


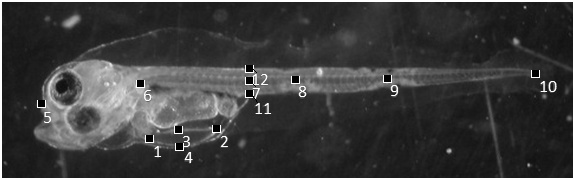

Supplement: Impacts of regular and random noise on the behaviour, growth and development of larval Atlantic cod (Gadus morhua) supplementary material [file rspb20151943supp1.docx]
